# RASAL1 is a potent regulator of hepatic stellate cell activity and liver fibrosis

**DOI:** 10.18632/oncotarget.17609

**Published:** 2017-05-04

**Authors:** Akemi Takata, Motoyuki Otsuka, Takahiro Kishikawa, Mari Yamagami, Rei Ishibashi, Kazuma Sekiba, Tatsunori Suzuki, Motoko Ohno, Yui Yamashita, Takaya Abe, Ryota Masuzaki, Tsuneo Ikenoue, Kazuhiko Koike

**Affiliations:** ^1^ Department of Gastroenterology, Graduate School of Medicine, The University of Tokyo, Tokyo, Japan; ^2^ Animal Resource Development Unit, RIKEN Center for Life Science Technologies, Kobe, Japan; ^3^ Genetic Engineering Team, RIKEN Center for Life Science Technologies, Kobe, Japan; ^4^ Division of Clinical Genome Research, Advanced Clinical Research Center, Institute of Medical Science, The University of Tokyo, Tokyo, Japan

**Keywords:** RASGAP, AGTR1, AMPK, SRF, MAPK, Pathology Section

## Abstract

Liver fibrosis, leading to cirrhosis and liver failure, can occur after chronic liver injury. The transition of hepatic stellate cells (HSCs) from quiescent cells into proliferative and fibrogenic cells is a central event in liver fibrosis. Here, we show that RAS protein activator like-1 (RASAL1), a RAS-GTPase-activating protein, which switches off RAS activity, is significantly decreased during HSC activation, and that HSC activation can be antagonized by forced expression of the RASAL1 protein. We demonstrate that RASAL1 suppresses HSC proliferation by regulating the Ras-MAPK pathway, and that RASAL1 suppresses HSC fibrogenic activity by regulating the PKA-LKB1-AMPK-SRF pathway by interacting with angiotensin II receptor, type 1. We also show that RASAL1-deficient mice are more susceptible to liver fibrosis. These data demonstrate that deregulated RASAL1 expression levels and the affected downstream intracellular signaling are central mediators of perpetuated HSC activation and fibrogenesis in the liver.

## INTRODUCTION

Liver fibrosis, observed clinically as liver cirrhosis, is a common consequence of chronic liver injury, such as that caused by chronic hepatitis viral infection (especially hepatitis B and C viruses), overload of alcohol or drugs, and steatosis. It is characterized by the progressive accumulation of extracellular matrix (ECM) proteins, such as type I and III collagens [1]. Hepatic stellate cells (HSCs), located in the peri-sinusoidal space of Disse, are a major source of ECM and play important roles in liver fibrosis [2]. In normal liver, HSCs are quiescent and store 80% of the total vitamin A in the body. After liver injury, HSCs are activated, characterized by loss of vitamin A, to transdifferentiate into smooth muscle α-actin (α-SMA)-positive myofibroblast-like cells [3]. Activated HSCs secrete cytokines and growth factors that increase their proliferation and production of ECM proteins [4]. The prolonged and repeated accumulation of ECM proteins disturbs the hepatic architecture by forming fibrotic scars and nodules, resulting in hepatic dysfunction [5].

Ras protein activator like 1 (RASAL1) is a member of the GTPase-activating protein (GAP) 1 family of GTPase-activating proteins. It acts as a suppressor of Ras function by enhancing the GTPase activity of Ras proteins, promoting conversion from the active form of Ras (GTP-bound Ras) to the inactive form (GDP-bound Ras). We previously detected frequent downregulation of RASAL1 expression in colon [6] and gastric cancers [7]. Later, others confirmed these findings and reported its downregulation in liver [8], bladder [9], and thyroid cancers [10] as well, suggesting that RASAL1 plays key roles in oncogenesis in various tissues, probably as a regulator of Ras function. In addition to its involvement in the pathogenesis of cancer, a genome-wide methylation screening using human fibrotic kidney fibroblasts and experimental renal fibrosis models identified that RASAL1 was hypermethylated via Dnmt1 after TGF-β stimulation in renal fibrosis, with substantial suppression of RASAL1 expression [11]. Hyperactivated Ras with reduced expression of RASAL1 in fibroblasts increased the intrinsic proliferative activity, type I collagen expression, and α-SMA expression of fibrotic fibroblasts [12, 13]. In addition, methyl-CpG-binding protein 2, which increases global methylation levels, has been reported to regulate RASAL1 expression in HSCs of liver fibrosis in rats [14]. Furthermore, in cardiac fibrosis, TGF-β causes aberrant methylation of the RASAL1 promoter, which was antagonized by BMP7 via TET3 in human coronary endothelial cells [15]. These results suggest that reduced RASAL1 expression is important for the pathogenesis of tissue fibrosis, in addition to its role in carcinogenesis. However, the molecular downstream events caused by deregulated RASAL1 expression levels have not been fully determined.

In the present study, we explored the role of RASAL1 in HSCs in *in vitro* studies as well as in RASAL1-knockout mice. The molecular analyses facilitated identification of the complex regulatory cascades linking the decreased expression of RASAL1 to the fibrotic activation of HSCs, which may contribute to the development of strategies for preventing liver fibrosis.

## RESULTS

### RASAL1 suppresses the activity of HSCs

**Figure 1:**
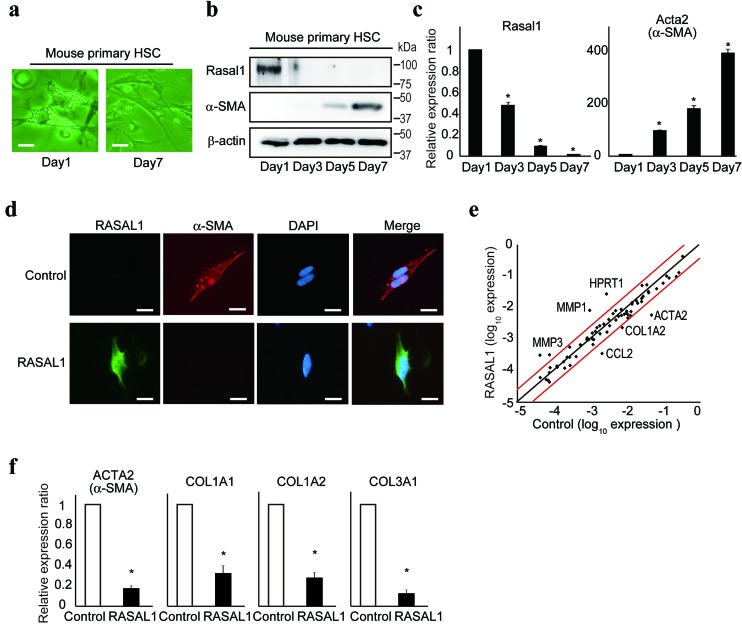
RASAL1 suppresses the activity of HSCs **a.**, Mouse primary HSCs were isolated and cultured on an uncoated plastic dish. Changes in the morphology of the cells are shown on days 1 and 7 of culture. Scale bar, 10 μm. **b.**, **c.**, Protein and transcript levels of α-SMA and RASAL1 in mouse primary HSCs during *in vitro* culture were assessed by Western blotting and quantitative RT-PCR, respectively. Representative results from two independent experiments are shown. **d.**, Protein levels of α-SMA in LX2 cells with forced RASAL1 expression were determined by immunocytochemistry. Representative results from five independent experiments are shown. Scale bar, 10 μm. **e.**, Scatter plot of the results of a fibrosis-related PCR array of control and RASAL1-expressing LX2 cells. Red lines indicate a twofold increase or 50% decrease in the expression level. Representative genes with significant changes in expression are indicated. A representative result of two independent experiments is shown. **f.**, mRNA levels of fibrosis-related genes were assessed by quantitative RT-PCR. Values are the mRNA levels in RASAL1-expressing cells relative to control LX2 cells. Data are means ± SD of three independent experiments. *, *p* < 0.05 (*t*-test).

Primary HSCs become activated spontaneously in *in vitro* culture [16]. To evaluate the expression levels of RASAL1 protein during the activation of HSCs, we cultured primary HSCs isolated from C57/B6 wild-type mice on an uncoated plastic dish to allow their spontaneous activation. Although lipid droplets reflecting vitamin A storage, which are characteristic of resting HSCs [17], were observed clearly in the cytoplasm of primary HSCs from mice on day 1 after isolation, they largely disappeared, and the cells showed a more spindle-like morphology on day 7 of culture, indicating spontaneous activation of HSCs *in vitro* (Figure 1a).

The expression levels of α-SMA increased over time in culture, which is characteristic of activated HSCs (Figure 1b). In contrast, the expression levels of RASAL1 were reduced dramatically (Figure 1b). The transcript levels of α-SMA and RASAL1 were correlated with their protein levels (Figure 1b, 1c). These phenomena were observed in independent experiments (Supplementary Figure 1a, b). *In vitro* studies showed that the expression levels of α-SMA protein decreased dramatically after forced expression of the RASAL1 protein in LX2 cells and in human activated and immortalized HSCs [18], as determined by Western blotting and immunohistochemistry (Supplementary Figure 1c and Figure 1d). Li-90, another human hepatic stellate cell line, showed similar results (Supplementary Figure 1d). Together, these results suggest that RASAL1 expression levels are inversely correlated with the activation of HSCs.

To confirm the biological effects of RASAL1 on the activity of HSCs, we next examined changes in the expression levels of fibrosis-related genes by forced RASAL1 expression in LX2 cells. The results of a PCR array showed decreased expression of a group of fibrosis-related genes, including ACTA2 (α-SMA), COL1A2, and CCL2, as well as increased expression levels of ECM degradative enzyme-related genes, such as matrix metalloproteinases (MMPs; Figure 1e). The simultaneously decreased mRNA levels of fibrosis-related genes, such as COL1A1, COL1A2, COL3A1, and ACTA2, by RASAL1 expression were confirmed by real-time PCR in LX2 and Li-90 cells (Figure 1f and Supplementary Figure 1e). Transcript levels of MMP-1 and MMP-3 were increased in RASAL1-expressing LX2 cells, similar to resting HSCs [19] (Supplementary Figure 1f). In contrast to the fibrotic liver, which expresses high levels of MMP-2 and MMP-9 [20], transcript levels of MMP-2 and MMP-9 were decreased in RASAL1-expressing LX2 cells (Supplementary Figure 1g). These results suggest that RASAL1 is a potent negative regulator of HSC activity.

### RASAL1 regulates RasGAP activity and HSC proliferation

**Figure 2:**
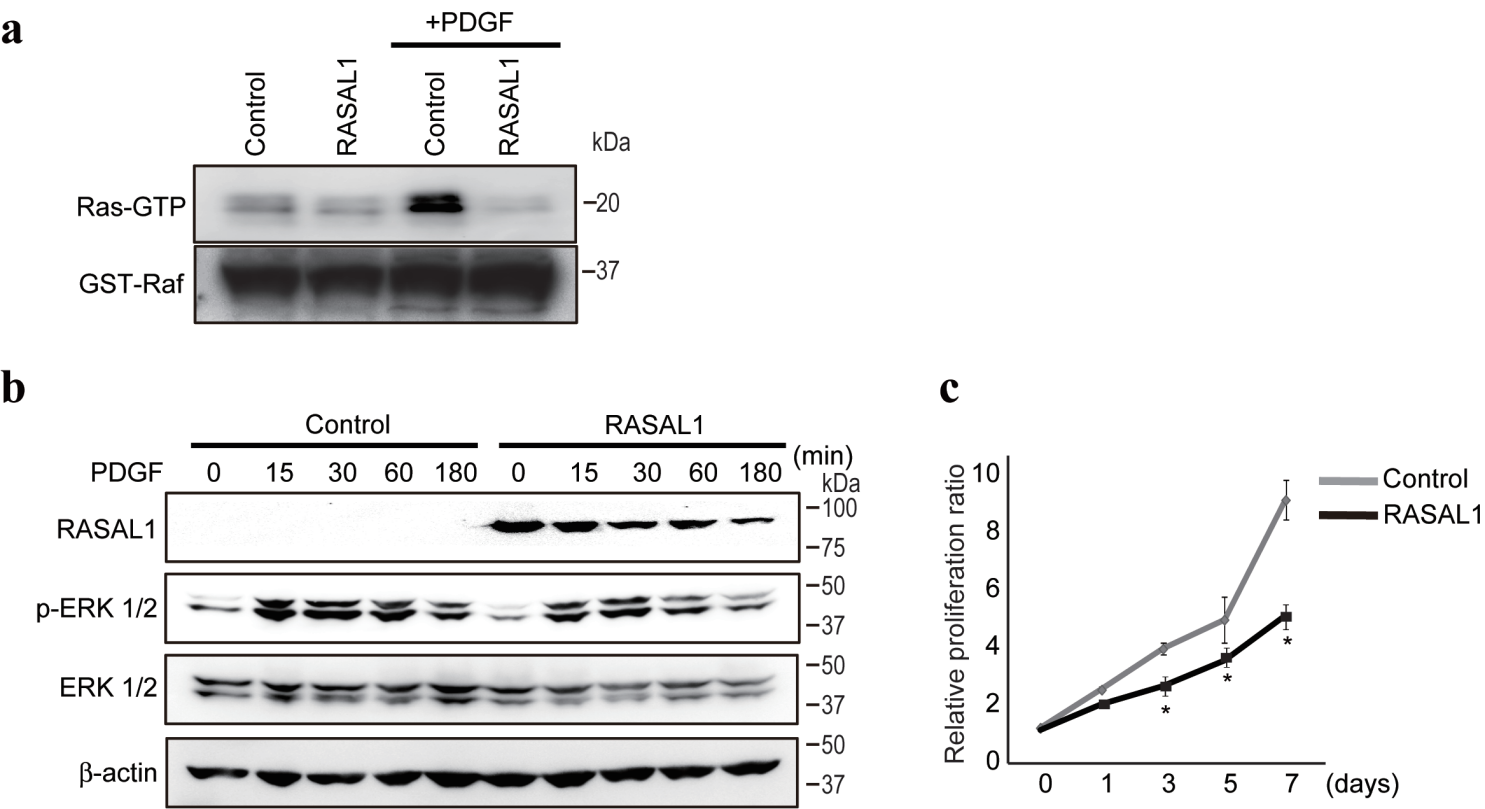
RASAL1 regulates RasGAP activity and proliferation in HSCs **a.**, Ras activity was determined by comparing the levels of active GTP-bound Ras (Ras-GTP) that bound to recombinant Raf protein in control and RASAL1-expressing LX2 cells, with or without PDGF treatment for 1 h as a positive regulator of Ras activity. Equal amounts of recombinant Raf protein in pull-down assays were confirmed by analyzing GST-Raf protein levels in the immunoprecipitates. Representative results of five independent experiments are shown. **b.**, The phosphorylation levels of Erk were lower in RASAL1-expressing cells compared with control LX2 cells after PDGF stimulation. A representative result from five independent experiments is shown. Similar results were obtained in Li-90 cells. **c.**, The proliferation rate of RASAL1-expressing cells was significantly lower than that of control LX2 cells after PDGF stimulation. The relative cell number was determined by adjusting the values obtained before PDGF treatment to 1. Data are means ± SD of three independent experiments. *, *p* < 0.05 (*t*-test).

Consistent with previous results that RASAL1 suppresses Ras function by promoting the conversion from the active to inactive form of Ras [21], Ras activity, determined by the degree of Ras-bound GTP binding to Raf, were impaired in LX2 cells by RASAL1 expression (Figure 2a). Platelet-derived growth factor (PDGF), a potent activator of the MAPK pathway in HSCs [22], activated Ras and induced the phosphorylation of Erk1/2 in control LX2 cells (Figure 2a, 2b). However, such activation was impaired significantly in LX2 cells with forced RASAL1 expression (Figure 2a, 2b). Consistently, although PDGF stimulation induced the proliferation of control LX2 cells, as described previously [22], the effects were markedly reduced in RASAL1-expressing cells (Figure 2c). These results suggest that RASAL1 expression in HSCs suppresses cell proliferation, as expected from the activation status of the Ras-MAPK pathway, as well as HSC activity and its role in fibrosis.

### RASAL1 suppresses HSC activation by inhibiting serum response factor (SRF) activity

Although activation of the Ras-MAPK pathway was significantly suppressed by RASAL1 expression, as described above, and the involvement of the MAPK pathway in HSC fibrotic activity has been reported [12], we did not observe a reduction in fibrotic activity in LX2 cells following MEK inhibitor treatment (Supplementary Figure 2a, 2b). Similarly, although Rho activity induces HSC activity [23], we did not observe any effect of RASAL1 expression on Rho activity in LX2 cells (Supplementary Figure 2c). These results suggested that the pathways are independent of the effects of RASAL1 on the fibrotic activity of HSCs.

**Figure 3:**
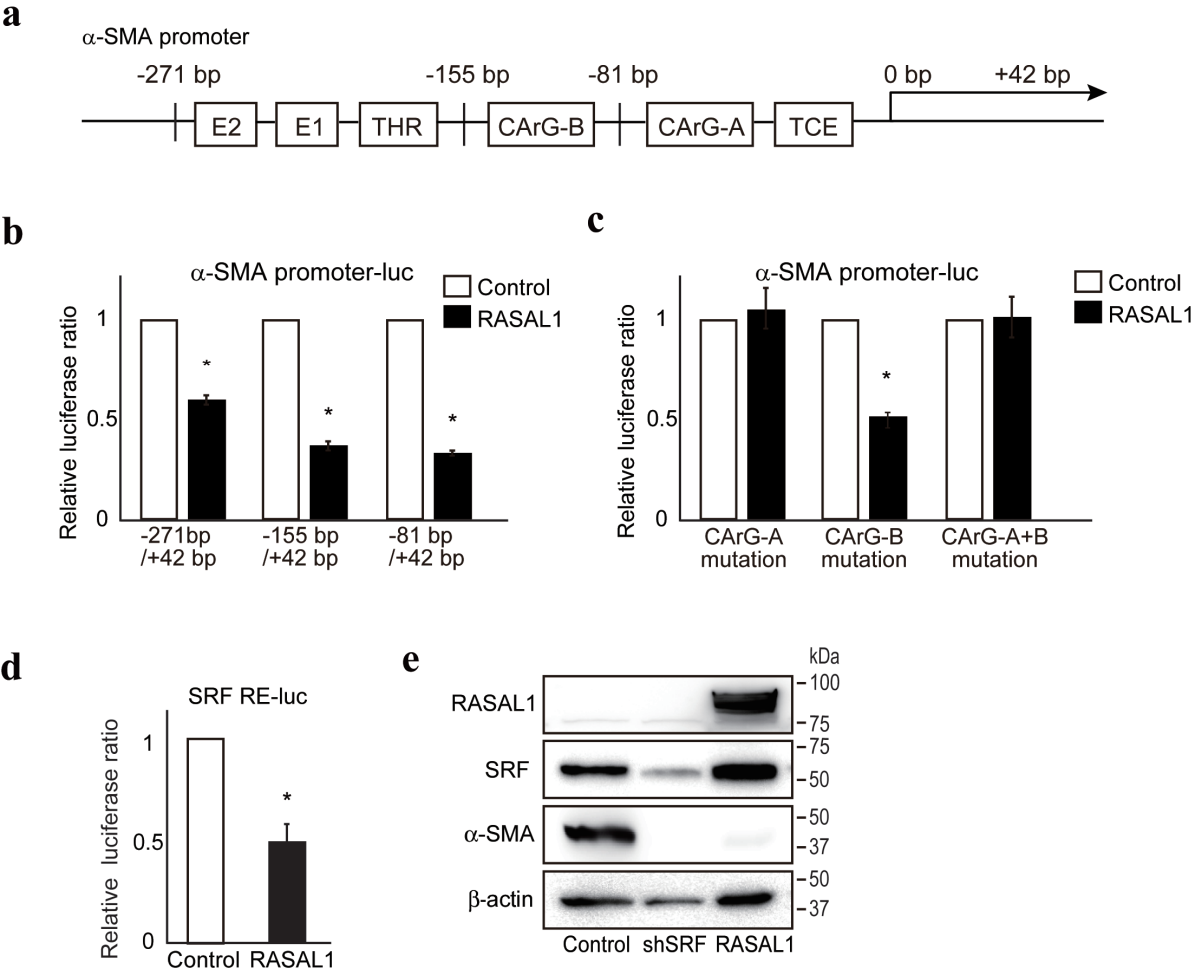
RASAL1 inhibits SRF activity **a.**, Schematic representation of the transcriptional regulatory regions in the α-SMA gene promoter. E1, E box 1 (CACCTG), E2, E box 2 (CACCTG), THR, TGF-β1 hypersensitivity region, CArG, SRF response element (CC(A/T)_6_GG), TCE, TGF-β1 control element. **b.**, Suppressive effects of RASAL1 expression (black bar) on α-SMA promoter activity. Test values were normalized to those obtained from cells transfected with an empty vector (white bar), which was set to 1. Data are means ± SD of three independent experiments in LX2 cells. *, *p* < 0.05 (*t*-test). **c.**, The CArG-A element was key for the suppressive effects of RASAL1 on α-SMA promoter activity. Reporter constructs with point mutations in the CArG elements (A, B, and A+B) were used. The results are shown as described in b. **d.**, RASAL1 suppressed SRF activity. Reporter constructs containing five SRF response elements in tandem (SRF RE-luc) were used to determine the effects of RASAL1 on SRF activity. Data are means ± SD of three independent experiments in LX2 cells. *, *p* < 0.05 (*t*-test). **e.**, LX2 cells with stable knockdown of SRF showed lower α-SMA protein levels. Representative results from three independent experiments are shown.

To define the molecular mechanism(s) underlying suppression of the fibrotic activity of HSCs by RASAL1, we examined the effects of RASAL1 on the promoter activity of α-SMA using reporter assays evaluating various α-SMA promoter deletion constructs. While RASAL1 expression showed significant suppression of promoter activity when a construct containing the α-SMA promoter region from −271 bp to +42 bp was used, such effects were similarly observed for constructs containing the region from −155 bp to +42 bp or from −81 bp to +42 bp (Figure 3a. b), suggesting that the −81 bp to +42 bp region of the promoter contains determinants of the suppressive effects of RASAL1 on α-SMA promoter activity. Because this region contains a CArG element, which is a target of SRF, a key transcription factor for α-SMA expression [24], we hypothesized that RASAL1 may have suppressive effects on the CArG-A element of the α-SMA promoter. No effect of RASAL1 was observed when using reporter constructs containing point mutations in the CArG-A element, which further supported our hypothesis (Figure 3c). Moreover, RASAL1 significantly suppressed the reporter activity of a construct that contained only five repeats of the SRF binding site (Figure 3d). Consistently, SRF knockdown suppressed the expression levels of α-SMA in LX2 cells (Figure 3e). Together, these results suggest that RASAL1 suppresses α-SMA expression in HSCs by inhibiting SRF activation.

### RASAL1 suppresses SRF activity through the PKA-LKB1-AMPK-MKL1 pathway

**Figure 4:**
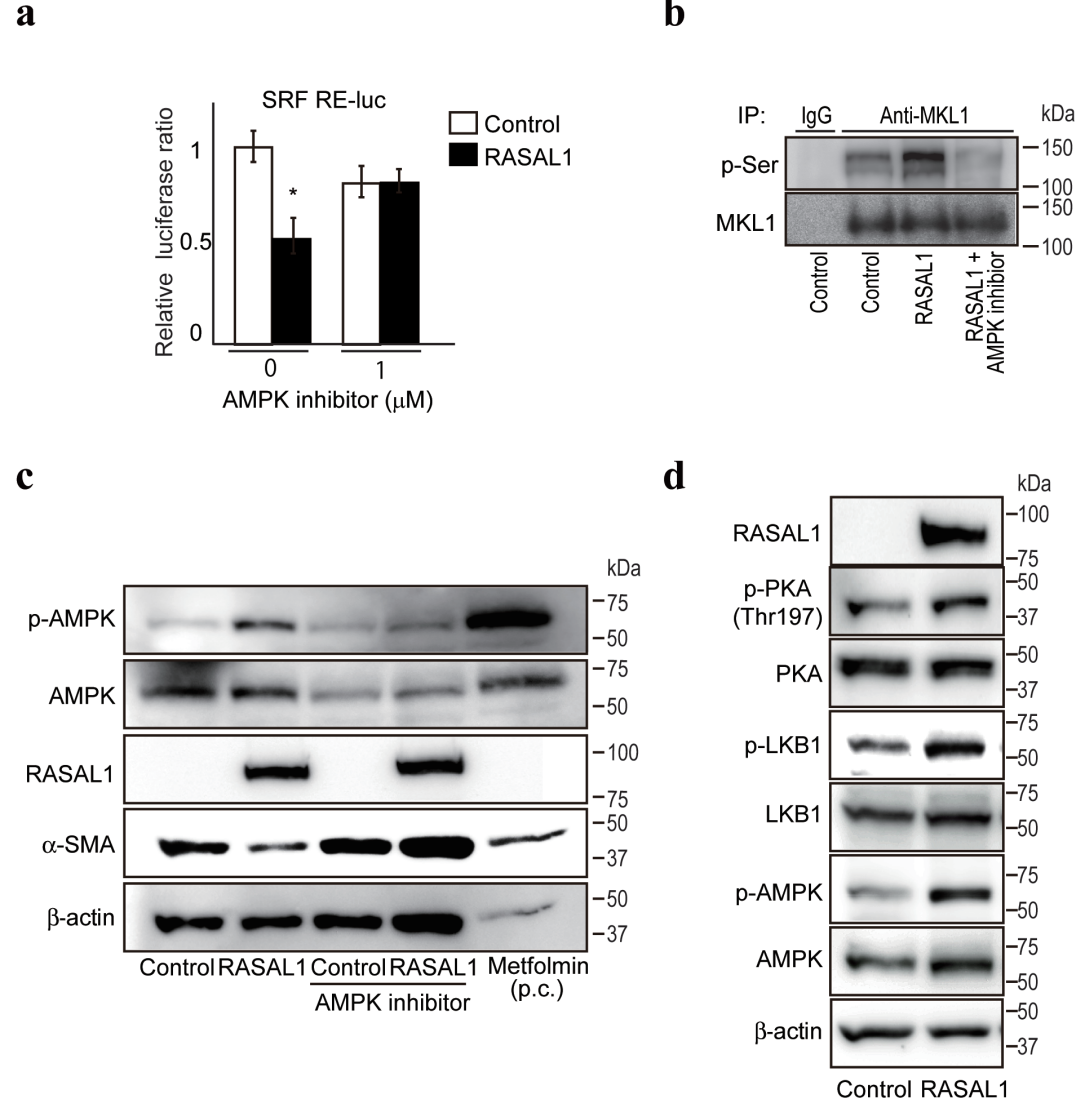
RASAL1 suppresses SRF activity via the PKA-LKB1-AMPK-MKL1 pathway **a.**, AMPK inhibitors restored the suppression of SRF **activity** by RASAL1. SRF RE-luc was used to determine the suppressive effects of RASAL1 on SRF **activity**. AMPK inhibitors were added for 24 h before the assay. Data are means ± SD of three independent experiments in LX2 cells. *, *p* < 0.05 (*t*-test). **b.**, Phosphorylation of MKL1 was increased by RASAL1 expression via AMPK activation. MKL1 proteins in LX2 control cells (control), RASAL1-overexpressing cells (RASAL1), and RASAL1-overexpressing cells after treatment with compound C for 6 h (RASAL1 + AMPK inhibitor) were immunoprecipitated. The phosphorylation status of MKL1 was determined by Western blotting using anti-phospho-serine antibodies. Immunoprecipitation using normal rabbit IgG was performed as a negative control. Representative results from three independent experiments are shown. **c.**, Effect of RASAL1 expression on the phosphorylation status of AMPK. Control and RASAL1-expressing LX2 cells were treated with AMPK inhibitor for 1 h. Metformin, an activator of AMPK, was used as a positive control (p.c.) and was added for 1 h to control LX2 cells. Cell lysates were subjected to Western blotting using antibodies against the indicated proteins. Representative results from three independent experiments are shown. **d.**, Phosphorylation of LKB1 and PKA was increased by RASAL1 expression. The phosphorylation and expression levels of the indicated proteins in control and RASAL1-expressing LX2 cells were determined. Representative results from three independent experiments are shown.

Next, to examine the molecular mechanism(s) by which RASAL1 suppressed SRF activity, we screened a library of 380 inhibitors in LX2 cells transiently co-transfected with SRF reporters and RASAL1-expressing plasmids to identify the pathways involved in the suppression of SRF activity by RASAL1. We found that compound C, a specific inhibitor of AMP-activated protein kinase (AMPK), restored SRF reporter activity (Figure 4a), suggesting that AMPK inhibits SRF activity. While no change in the phosphorylation status of SRF was observed under AMPK inhibitor treatment (Supplementary Figure 3), phosphorylation of the SRF coactivator megakaryoblastic leukemia 1 (MKL1), which affects SRF activity negatively [25], was induced by RASAL1 expression and was antagonized by an AMPK inhibitor (Figure 4b). These results suggested that the negative effects of AMPK on SRF activity are induced by RASAL1 expression, and that these effects are canceled by an AMPK inhibitor. Consistently, AMPK inhibitors canceled the suppressive effects of α-SMA expression by RASAL1 in LX2 cells (Figure 4c). Moreover, consistent with the possible involvement of AMPK in the effects of RASAL1, AMPK phosphorylation was increased by RASAL1 expression (Figure 4c). Furthermore, the degree of phosphorylation of the serine‒threonine kinase liver kinase B1 (LKB1, also known as STK11), a master kinase that phosphorylates and activates AMPK [26], was increased significantly in RASAL1-expressing LX2 cells (Figure 4d). Moreover, cAMP-dependent protein kinase A (PKA), which phosphorylates LKB1 [27], was also phosphorylated to a greater degree in RASAL1-expressing LX2 cells (Figure 4d). Together, these results suggested that the suppressive effects of RASAL1 on SRF activity are mediated, at least in part, by activation of the PKA-LKB1-AMPK-MKL1 signaling pathway.

### RASAL1 inhibits Gαi activity coupled with angiotensin II receptor, type 1 (AGTR1)

**Figure 5:**
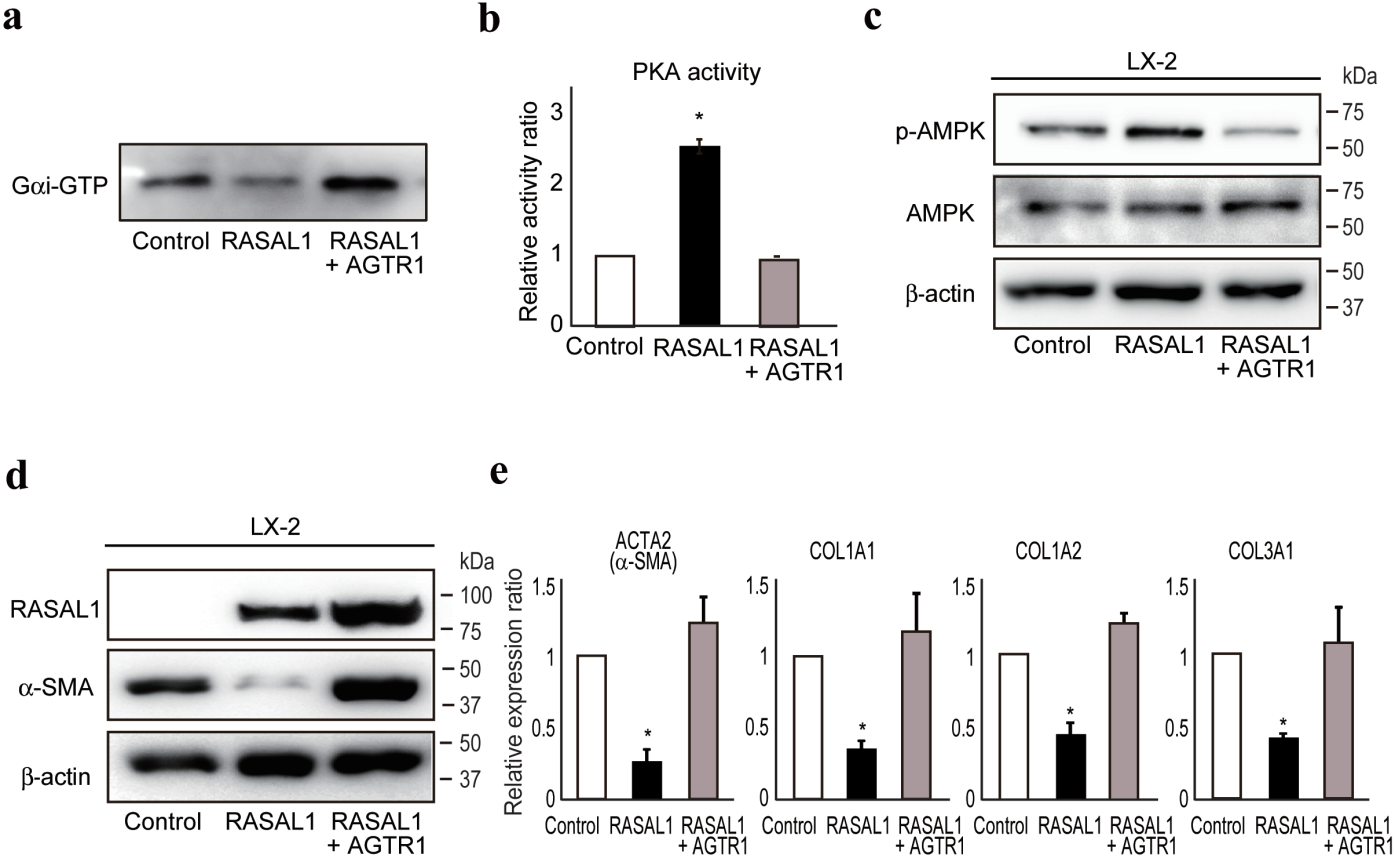
RASAL1 interacts with AGTR1 and regulates Gαi function **a.**, Gαi activity in control (Control), RASAL1-expressing (RASAL1), and RASAL1- and AGTR1-expressing (RASAL1 + AGTR1) LX2 cells subjected to GTP-bound active Gαi pull-down assay. A representative result from five independent experiments is shown. **b.**, PKA activity in control (Control), RASAL1-expressing (RASAL1), and RASAL1- and AGTR1-expressing (RASAL1 + AGTR1) LX2 cells measured by ELISA using cell lysates and phosphorylation-specific antibodies against PKA substrate peptide. Data are means ± SD of three independent experiments. *, *p* < 0.05 (*t*-test). **c.**, **d.**, The levels of phosphorylated AMPK (c) and α-SMA protein (d) in control, RASAL1-expressing, and RASAL1- and AGTR1-expressing LX2 cells were determined by Western blotting. **e.,** Expression levels of fibrosis-related gene transcripts were assessed by quantitative RT-PCR. Values are the mRNA expression levels in RASAL1-expressing cells and RASAL1- and AGTR1-expressing cells relative to those in control LX2 cells. Data are means ± SD of three independent experiments. *, *p* < 0.05 (*t*-test).

Angiotensin II is a potent inducer of liver fibrosis, as determined by experimental and clinical observations [28-30], and AGTR1 was recently implicated in the development of liver fibrosis via activation of JAK2 [31]. Because the angiotensin II receptor can inhibit PKA [32, 33], to determine further how RASAL1 increases the phosphorylation of PKA and suppresses the downstream fibrotic activity of HSCs, we next examined the interaction between RASAL1 and AGTR1. We found that RASAL1 potently interacted with AGTR1 (Supplementary Figure 4a). AGTR1 interacts with heterotrimeric G proteins, composed of three G-protein subunits, including Gαi and Gαq, which are coupled with distinct signaling cascades [34, 35]. Gαi, coupled with the βγ subunit, is responsible for inhibition of adenylyl cyclase activity, resulting in a decrease in PKA activity. Gαq activates phospholipase C, which hydrolyzes phosphatidylinositol 4, 5-bisphosphate to inositol triphosphate and diacyl glycerol, which activates protein kinase C (PKC). While similar PKC activity was observed between control and RASAL1-expressing LX2 cells (Supplementary Figure 4b), we found that RASAL1 inhibited Gαi activity, which was antagonized by forced overexpression of AGTR1 (Figure 5a). PKA activity and the subsequent phosphorylation of AMPK were enhanced by RASAL1 expression, which was antagonized by AGTR1 expression (Figure 5b, 5c). The suppressive effects of RASAL1 on **α**-SMA protein levels and the mRNA levels of other fibrosis-related factors in LX2 cells were antagonized significantly by AGTR1 overexpression (Figure 5d, e). Although phosphorylation of JAK2 was recently reported to be involved in liver fibrosis downstream of AGTR1-mediated signaling in HSCs [31], RASAL1 had no effect on phosphorylation of JAK2 (Supplementary Figure 4c). RASAL1 had no effect on TGF-β signaling activation, as determined by the similar degree of Smad2 nuclear import between control and RASAL1-expressing LX2 cells (Supplementary Figure 4d). While angiotensin II-induced Erk2 phosphorylation was suppressed by RASAL1, AGTR1 overexpression had no rescue effect, in contrast to its fibrosis-related effects, suggesting that RASAL1 suppressed activation of the MAPK pathway downstream of AGTR1, possibly at points distinct from the fibrosis-related pathway (Supplementary Figure 4e). These results indicated that RASAL1 interacts with AGTR1 and inhibits Gαi activity coupled with AGTR1, which, in turn, suppressed the activity of HSCs.

### RASAL1-deficient mice are more susceptible to liver fibrosis

**Figure 6:**
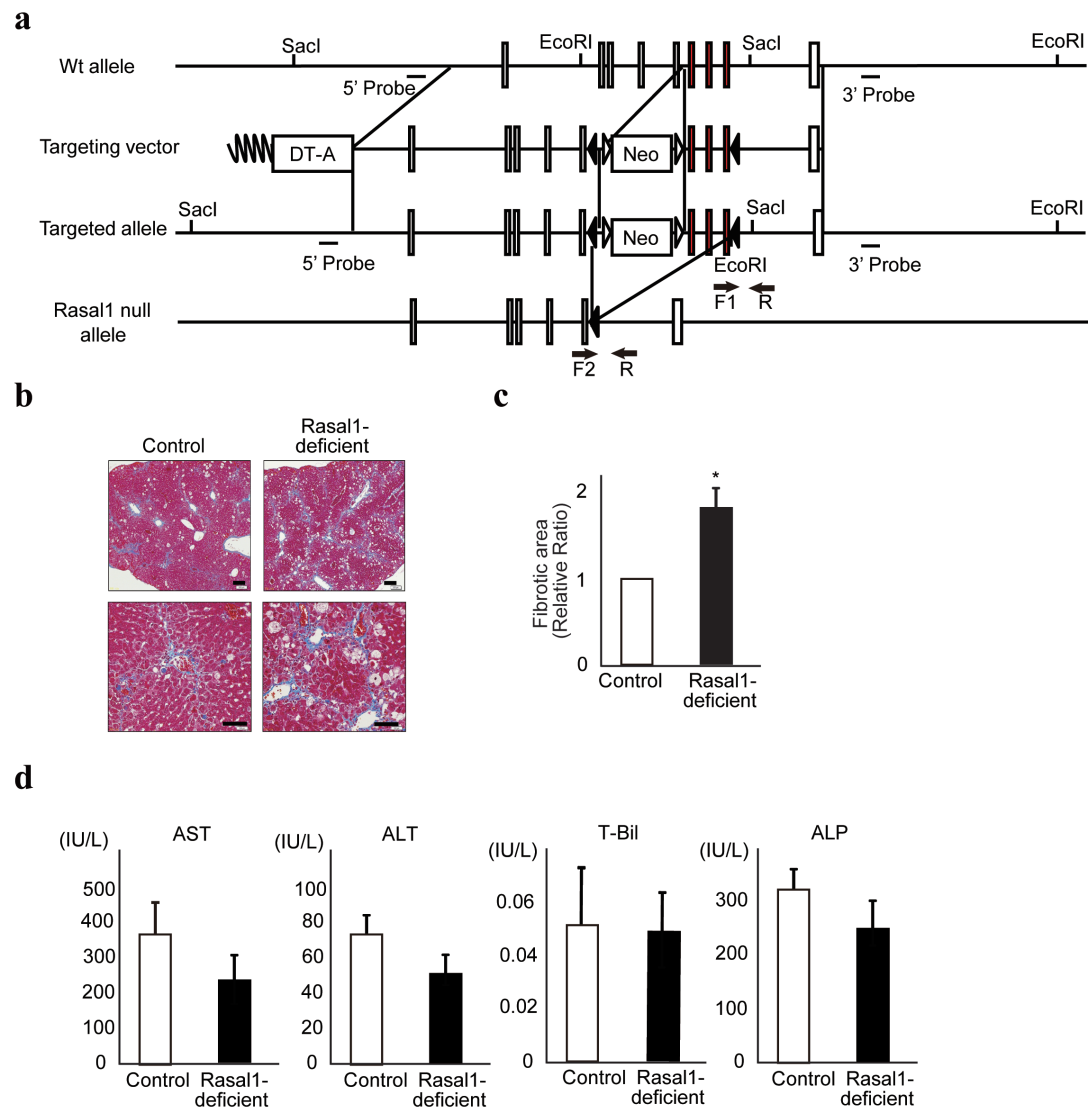
RASAL1-deficient mice are more susceptible to liver fibrosis **a.**, Generation of the *Rasal1* null allele. After Cre-mediated recombination, exons 11‒13 and the Neo cassette were deleted, resulting in a *Rasal1* null allele. Exons 11‒13 are denoted by red boxes. The other exons are denoted by open boxes. Solid triangles represent *lox*P sites. Open triangles represent FRT sites. Arrows indicate the positions of the primers used for genotyping (F1, F2, and R). **b.**, Histological images of the liver stained with Masson’s trichrome after induction of experimental liver fibrosis using CCl_4_ are shown. While both control and RASAL1-deficient mice exhibited liver fibrosis (blue staining), the degree of fibrosis was significantly greater in the RASAL1-deficient mice. A representative result from four mice per group is shown. Scale bar, 500 μm. **c.**, Quantitative liver fibrotic areas in control and RASAL1-deficient mice, determined using ImageJ software. Data are means ± SD of four mice per group. *, *p* < 0.05 (*t*-test). **d.**, The degree of liver inflammation was similar between control and RASAL1-deficient mice. Biochemical parameters reflecting liver inflammation were measured in sera at 48 h after the final CCl_4_ injection. Data are means ± SD of four mice per group. *, *p* < 0.05 (*t*-test).

Finally, to determine the biological roles of RASAL1 in liver fibrosis *in vivo***,** we generated RASAL1-deficient mice by targeting the RASAL1 gene (Figure 6a and Supplementary Figure 5a). While we expected spontaneous fibrosis in these mice, this was not observed. However,

RASAL1-deficient mice showed more CCl_4_-induced liver fibrosis [36] than did wild-type littermate mice, as indicated by the percentage of fibrotic area according to Masson’s trichrome staining (Figure 6b, 6c)**.** Because liver damage was comparable between wild-type and RASAL1-deficient mice under this condition, as indicated by similar serum transaminase levels and bilirubin levels (Figure 6d), the difference in the degree of fibrosis was not due to a difference in the degree of inflammation. Similar results were obtained with fibrotic stimulation by TAA, which indicated more fibrosis in RASAL1-deficient mice with similar serum transaminase levels and bilirubin levels compared with wild-type mice (Supplementary Figure 5b, c, and d). These results suggested that RASAL1-deficient mice are susceptible to liver fibrosis, consistent with the observed effects *in vitro*.

Lastly, RASAL1 protein expression levels of HSCs in human pathological liver tissues were examined by immunohistochemistry. While RASAL1 protein is expressed in resting HSCs (expressing vimentin) as well as hepatocytes (Supplementary Figure 6a), its expression was significantly decreased in activated HSCs (expressing SMA) in fibrotic liver tissues (Supplementary Figure 6b and c).

## DISCUSSION

In this study, we showed that downregulated expression of RASAL1 in HSCs is characteristic of HSC activation in the liver. In addition, we showed by *in vitro* and *in vivo* studies that the expression of RASAL1 functionally regulated HSC activity. We found that RASAL1 binds to AGTR1. Reduced expression levels of RASAL1 increased Gαi activity downstream of AGTR1, which, in turn, suppressed PKA activity. Decreased PKA activity resulted in decreased phosphorylation of LKB1 and AMPK, suppressing inhibitory SRF phosphorylation, which is important for the expression of fibrotic proteins such as α-SMA, COL1A1, and COL1A2. This AGTR1-PKA-AMPK-SRF pathway in liver HSCs may contain novel targets for anti-fibrotic therapy.

RASAL1 was originally identified as a transcriptional target of the pituitary transcription factor PITX1, which suppresses Ras activity [37]. The suppression of Ras activity by PITX1 was mediated by increased expression of the GAP RASAL1, which hydrolyses GTP bound to active RAS, attenuating RAS activity. We previously found that RASAL1 expression was specifically downregulated in colon cancers containing wild-type Ras [6] and in gastric carcinoma [7], and this may be a mechanism of inducing wild-type Ras oncogenic activity. The activation of HSCs may occur during carcinogenesis in epithelial cells, along with activation of the Ras-related pathway, consistent with a recent report [38]. Growth advantages were, in fact, observed in activated HSCs with decreased RASAL1 expression in this study.

However, the fibrotic activity of active HSCs was not dependent on the Ras-MAPK pathway. Rather, RASAL1 affected the AGTR1-PKA-AMPK-SRF pathway by binding to AGTR1, which is critical for the expression of α-SMA and collagens in activated HSCs. While our finding that RASAL1 also suppressed receptor-coupled G protein by binding to AGTR1 was unexpected, these results may be similar to RGS14 modulation of intracellular signaling by acting as a GAP both for the Gαi subunit and for small GTPases [39]. Neurofibromin also regulates both receptor-coupled Gα and small GTPases [40]. RASAL1 may function similarly to these RasGAP-like proteins, although the precise mechanisms underlying RASAL1 regulation of small G proteins coupled with cell surface receptors and its specificity remain to be determined.

We demonstrated that the AMPK-MKL1-SRF pathway, downstream of PKA, is important for fibrotic activation of HSCs with decreased RASAL1 expression. We also identified by screening that compound C, an inhibitor of AMPK, activates SRF activity by antagonizing the suppressive effects of RASAL1. Clinically, AMPK-activating compounds, such as metformin, are widely used for their beneficial effects on glucose metabolism. The benefit of metformin in improving the survival of patients with liver cirrhosis was recently confirmed clinically [41]. Our results regarding the pathway of AMPK-mediated suppression of SRF and HSC activity support such beneficial effects of metformin on patients with liver cirrhosis.

Another important finding was that the effects of RASAL1 in HSCs were mediated by AGTR1. Angiotensin II and AGTR1 interactions may play a pivotal role in liver fibrosis [29, 42]. In fact, angiotensin-converting enzyme inhibitors and AT1-R blockers have been reported to improve liver fibrosis scores clinically [43], suggesting that the AGTR1-mediated pathway is involved in HSC activation. Our data on the effects of RASAL1 on AGTR1 with respect to HSC fibrotic activity are consistent with these clinical data. The effects of targeting these pathways on liver fibrosis need to be evaluated further in randomized, controlled clinical trials. Additionally, because the renin-angiotensin system is involved in the development of fibrosis in many tissues as a result of AGTR1 stimulation [44, 45], deregulated expression of RASAL1 may also be involved in fibrosis in other tissues.

Genetically modified RASAL1-deficient mice were generated in this study to confirm the biological roles of RASAL1 observed *in vitro*. Whereas significant spontaneous liver fibrosis was not observed in these mice, RASAL1-deficient mice showed exacerbated fibrosis after chemical induction of liver fibrosis. While fibrotic induction was needed to observe clear differences, this was probably because rodents are generally resistant to spontaneous fibrosis [46]. Additionally, no pathological spontaneous tumors were seen in these mice up to 48 weeks of age under a normal diet and conditions.

Liver fibrosis is clinically recognized as a high-risk factor for the incidence of hepatocellular carcinoma. One possible reason is suggested by the elegant concept of senescence of activated HSCs, which produce senescence-associated secretory phenotype factors that affect non-cell autonomous functions, such as tumorigenesis, in hepatocytes [47]. Because treatment with DMBA, a chemical carcinogen that activates oncogenic Ras, was used during the neonatal stage in parallel with feeding a high-fat diet for 30 weeks, it will be interesting to determine whether similar tumorigenic phenomena can be obtained in our RASAL1-deficient mice, which also harbor activated Ras and activated HSCs. Ongoing characterization of these mice will be required to identify the physiological roles of RASAL1 with respect to tumorigenesis.

Downregulated expression of RASAL1 in epithelial cells and fibroblast are mostly mediated by methylation in its promoter regions [11, 14]. Although we did not investigate the mechanisms of the downregulation of RASAL1 expression in HSCs in this study, it will be crucial to determine whether similar promoter methylation is involved in the downregulation of RASAL1 expression in HSCs as well in the future.

In summary, we have shown that RASAL1 plays pivotal roles in the regulation of the proliferation and fibrotic activity of HSCs. These effects were mediated by two distinct pathways: the Ras-MAPK and AGTR1-AMPK-SRF pathways. These intracellular signaling pathways in liver HSCs may represent novel targets for anti-fibrotic therapy in patients with liver cirrhosis progression.

## MATERIALS AND METHODS

### Mouse experiments

Mouse experimental protocols were approved by the Ethics Committee for Animal Experimentation at the University of Tokyo (#13-P-54). The experiments were conducted in accordance with the guidelines for the care and use of laboratory animals of the University of Tokyo.

### RASAL1-deficient mice

To generate *Rasal1*-deficient mice (accession no. CDB0814K: http://www.clst.riken.jp/arg/mutant%20mice%20list.html), we designed a targeting vector to disrupt exons 11‒13 of the mouse *Rasal1* gene, which encode the majority of the RasGAP domain. In the targeting vector, two *lox*P sites were introduced at positions upstream of exon 11 and downstream of exon 13 of *Rasal1*. The floxed arm containing exons 11‒13 and two homology arms upstream of exon 11 and downstream of exon 13 were 1.1, 5.9, and 1.5 kb in length, respectively. They were obtained from a bacterial artificial chromosome clone containing the mouse *Rasal1* gene (RP23-323H12, BACPAC Resources) and assembled into a vector containing two *lox*P and FRT sites, the neomycin-resistance gene (Neo) cassette, and the diphtheria toxin fragment A cassette. Gene targeting was performed in the TT2 ES cell line [48]. Homologous recombinant clones were obtained from G418-resistant colonies. Correctly targeted ES cell clones were identified by Southern blotting using the indicated 5’ and 3’ probes. Positive clones were injected into eight-cell-stage ICR mouse embryos. Resulting male chimeras were bred with C57BL/6J females to achieve germline transmission of the targeted *Rasal1* allele. Heterozygotes were bred with congenic C57BL/6J transgenic mice expressing the Cre recombinase under the control of the chicken β-actin promoter (CAG-Cre) to delete both the Neo cassette and exons 11‒13 for generation of a null allele. The primer sequences used for genotyping were F1 (for the floxed allele), CAA GAT GGA CCT GAA CCG CTC T; F2 (for the knockout allele), GCC TCT CAT GGA ACT GCT TCT GGA G; and R (for the floxed and knockout alleles), TCC TCC TTG TCT GAG CTT TAC AT. The product sizes were 358, 300 bp, and 342 bp for the floxed, wild-type (using the F1 and R primers), and knockout (using the F2 and R primers) sequences, respectively.

### Liver fibrosis *in vivo*

Six-week-old Rasal1-deficient mice and their littermates were injected intraperitoneally with 1 mL/kg carbon tetrachloride (CCl4; Wako Chemicals, Osaka, Japan) dissolved in corn oil twice per week for 8 weeks. As another liver fibrosis model, 200 mg/kg thioacetamide (TAA; Wako) were injected into mice three times per week for 8 weeks. Mice were sacrificed 2 days after the last injection. Sera were collected, and biochemical parameters were measured at a clinical laboratory testing company (SRL, Inc., Tokyo, Japan). Liver tissues were serially sectioned and stained with Masson’s trichrome. Images were captured using the DP2-BSW software (Olympus, Tokyo, Japan), and the fibrotic areas were measured using the ImageJ software (NIH; http://rsbweb.nih.gov/ij/index.html).

### Primary HSC isolation

Primary HSCs were isolated from mice using collagenase/pronase digestion and OptiPrep (Nycomed, Oslo, Norway) with gradient centrifugation, as described previously [49]. Briefly, after the abdomen of each mouse was opened under anesthesia, cannulas were inserted into the portal vein (inflow) and inferior vena cava (outflow). The liver was perfused continuously with HBSS containing EGTA, 0.2% pronase E, and 0.05% collagenase for 10 min at 37°C until the liver color changed from red to loam. The liver was removed from the abdominal cavity and placed in a sterile dish. The liver tissue was minced and stirred at 37°C in HBSS containing 0.02% pronase E and DNase. The tissues were filtered through a nylon mesh and washed three times in DMEM containing DNase, followed by centrifugation (1600 rpm, 7 min). The cell suspension was resuspended in 11.5% OptiPrep and transferred to a 17.6% OptiPrep-containing tube. Then, 4 mL HBSS were carefully added onto the 11.5% OptiPrep containing the cell suspension and centrifuged (2,800 rpm, 14 min, 0°C). The cell layer between the HBSS and 11.5% OptiPrep was collected carefully; these cells were considered purified mouse primary HSCs. The cells were washed in DMEM containing DNase, followed by centrifugation (1,600 rpm, 5 min, 37°C). The isolated cells were seeded into uncoated plastic tissue-culture dishes (Falcon, Lincoln Park, NJ, USA) in DMEM supplemented with 10% FBS.

### *In vitro* studies

The *in vitro* experimental methods are described in the Supplementary Information.

### Statistical analysis

Results are expressed as means ± standard deviation (SD). Statistical evaluations were performed using Student’s t-test. Values of *p* < 0.05 were considered to indicate statistical significance.

## SUPPLEMENTARY MATERIALS FIGURES



## Abbreviations

HSC: hepatic stellate cell
RASAL1: RAS protein activator like-1
GAP: GTPase-activating protein
AGTR1: angiotensin II receptor, type 1
ECM: extracellular matrix
MMP: matrix metalloproteinase
SMA: smooth muscle actin
PDGF: Platelet-derived growth factor
SRF: serum response factor
AMPK: AMP-activated protein kinase
MKL1: megakaryoblastic leukemia 1
LKB1: liver kinase B1
PKA: cAMP-dependent protein kinase
PIP2: phosphatidylinositol 4, 5-bisphosphate
PKC: protein kinase C
DAG: diacyl glycerol
IP3: inositol triphosphate
TAA: thioacetamide
GAP: GTPase-activating protein
ARB: AT1-R blocker
SASP: senescence-associated secretory phenotype
