# Supplementary material for: RASAL1 is a potent regulator of hepatic stellate cell activity and liver fibrosis
Source: Oncotarget. 2017 May 4;8(39):64840–52. doi: 10.18632/oncotarget.17609 (PMC5630295; doi:10.18632/oncotarget.17609)
Supplement: Supplementary file 1 [file oncotarget-08-64840-s001.pdf]

www.impactjournals.com/oncotarget/ Oncotarget, Supplementary Material

RASAL1 is a potent regulator of hepatic stellate cell activity and liver fibrosis

Supplementary Materials

Supplementary Experimental Procedures

Supplementary References

Supplementary Figure Legends

Supplementary Figures (Figures S1-S5)

SUPPLEMENTARY EXPERIMENTAL PROCEDURES

Primers

The primers used (5'-3') were: RASAL1 forward, TGG ATT TCT CTT CTT GCG ATT CT, RASAL1 reverse, TGT TGG TCC CGA AGG TCA A, α-SMA forward, CCG ACC GAA TGC AGA AGG: α-SMA reverse, ACA GAG TAT TTG CGC TCC GGA, COL1A1 forward, CAT TGT TTC CTG TGT CTT CTG G, COL1A1 reverse, ACA GAG TAT TTG CGC TCC GGA, COL1A2 forward, AGG GCA ACA GCA GGT TCA CTT ACA, COL1A2 reverse, AGC GGG GGA AGG AGT TAA TGA AAC, COL3A1 forward, CAC GGA AAC ACT GGT GGA CAG ATT, COL3A1 reverse, ATG CCA GCT GCA CAT CAA GGA C, MMP1 forward, AGC TAG CTC AGG ATG ACA TTG ATG, MMP1 reverse, GCC GAT GGG CTG GAC AG, MMP3 forward, TGG CAT TCA GTC CCT CTAT GG, MMP3 reverse, AGG ACA AAG CAG GAT CAC AGT T, MMP2 forward, ACG ACC GCG ACA AGA AGT AT, MMP2 reverse, ATT TGT TGC CCA GGA AAG TG, MMP9 forward, GAC AAG CTC TTC GGC TTC TG, MMP9 reverse, CTC GCT GGT ACA GGT CGA GT, AGTR1 forward, ATT TAG CAC TGG CTG ACT TAT GC, AGTR1 reverse, CAG CGG TAT TCC ATA GCT GTG, GAPDH forward, ATC AAC GAC CCC TTC ATT GAC C, GAPDH reverse, CCA GTA GAC TCC ACG ACA TAC TCA GC, GAPDH reverse, CCA GTA GAC TCC ACG ACA TAC TCA GC, mouse Rasal1 forward, ACC AAG CTG GTG AAG CTG TT, mouse Rasal1 reverse, CAT CTT GCA AGG GTC CAG TT, mouse Gapdh forward, CCC CAC TAA CAT CAA ATG GG, mouse Gapdh reverse, ATC CAC AGT CTT CTG GGT GG, mouse Acta2 (α-SMA) forward, CTC TTC CAG CCA TCT TTC ATT G, mouse Acta2 (α-SMA) reverse, GTT GTT AGC ATA GAG ATC CTT CC.

Cell culture

The human hepatic stellate cell line LX2 was kindly provided by Dr. Friedman (1). The human hepatic stellate cell line Li-90 was obtained from the Japanese Collection of Research Bioresources (JCRB, Osaka, Japan). 293T cells were obtained from the American Type Culture Collection (ATCC, Rockville, MD). Cells were cultured in DMEM supplemented with 10% FBS and antibiotics.

Reagents

Human TGF-β1 was purchased from Pepro Tech Inc. (Rocky Hill, NJ). Human PDGF-BB was from the Cosmo Biologicals (Tokyo, Japan). PD98059, a MEK inhibitor, was from Daiichi Pure Chemicals (Tokyo, Japan). Nocodazole, angiotensin II, and metformin were from Wako Chemicals. Compound C, an AMPK inhibitor, was purchased from Calbiochem (Billerica, MA).

Antibodies

Human RASAL1 antibody was generated as described previously (2). An anti-β-actin antibody was obtained from Sigma-Aldrich (St. Louis, MO). The alpha smooth muscle actin (α-SMA) antibody was from Dako (Carpinteria, CA). Phosphorylated Erk1/2 (Thr 202/Thr 204), phospho-SRF (Ser 103), phospho-AMPK-alpha (Thr 172), phospho-LKB1 (Ser 428), phospho-PKA C (Thr 197) phospho-JAK2 (Tyr 1007/Tyr 1008), phospho-Ser, phospho-Thr, total Erk1/2, SRF, AMPK-alpha, LKB1, JAK2, MKL1, and PKA C-alpha antibodies were purchased from Cell Signal Technology (Beverly, MA). The Smad2/3 antibody was from BD Transduction Laboratories

(Lexington, KY). Mouse RASAL1 antibodies were obtained from Santa Cruz Biotechnology (Santa Cruz, CA). The Halo-tag antibody was purchased from Promega. The Flag (DYKDDDDK)-tag antibody was purchased from Wako.

Immunoprecipitation

For immunoprecipitation, cell lysates were precipitated by incubation with anti-FLAG M2 agarose (Sigma) for 8 h, or by incubation with anti-MKL1 or anti-SRF antibodies, followed by addition of protein A/G agarose (Santa Cruz) overnight. Cell extracts were prepared as described previously (3).

Western blotting

Western blotting was conducted as described previously (3).

Plasmids

The human RASAL1 expression plasmid (pcDNA3.1-RSAL1) was constructed as described previously (2). RASAL1 cDNA was subcloned into pCDH-puromycin vector (System Biosciences, Mountain View, CA) for lentivirus production by an infusion method (Clontech) at *Eco*RI site using the following primers: 5’-TAG AGC TAG CGA ATT CAC CTG AGC GCC CTT CTG C-3’ and 5’-ATT TAA ATT CGA ATT CCC TGG CTC TTG CTC CTC C-3’. For Flag-tagged RASAL1-expressing plasmid construction, the primers used were 5’-ATC GGA TCC GCG GCC GCG CCG CCA TGG ACT ACA AGG ACG ACG ACG ACA AGG ACT ACA AGG ACG ACG ACG ACA AGA TGG CCA AGA GCA GCT C-3’ and 5’-AGA TCC TTC GCG GCC GCA TTT CCT TAG GGG CCA AGG G-3’. The Halo-tagged AGTR1 clone was purchased

from Promega. PCR-amplified AGTR1 cDNA from the Halo-tagged AGTR1 clone was inserted into the pCDH-neomycin vector (System Biosciences) at the *Not*I site to produce AGTR1-expressing lentiviruses. The primers used were 5'-ATC GGA TCC GCG GCC GCA CCA TGA TTC TCA ACT CTT CTA C-3' and 5'-CAG ATC CTT GCG GCC GCT CAC TCA ACC TCA AAA CAT GG-3'. The mouse RASAL1-expressing plasmid was constructed by cloning of mouse RASAL1 cDNA into the pcDNA3-HA vector.

Lentivirus production and transduction

cDNA-expressing lentiviruses were produced by transfection of a cDNA-expressing pCDH vector and pPACKH1 packaging plasmid mix (System Biosciences) into 293T cells. After 48 h, virus-containing supernatants were collected and viral particles were concentrated using PEG-it Virus Precipitation Solution according to the manufacturer's protocol (System Biosciences). To generate stable RASAL1- and AGTR1-overexpressing cells, LX2 cells were transduced with RASAL1- or AGTR1-expressing constructs containing lentiviral particles, followed by selection with 2 μg/mL puromycin or 600 μg/mL neomycin.

Reporter assays

Dual reporter assays using pGL4-TK, a Renilla luciferase construct as a control, were performed basically as described previously (4). The series of α-SMA promoter-driven reporters was kindly provided by Dr. Manabe (5). SRF-RE luc, containing a firefly luciferase gene driven by a TATA box plus five repeats of the binding sites for SRF (GTCCATATTAGGAC), was obtained from Stratagene (La Jolla, CA). An AGTR1

promoter-driven reporter construct was purchased from GeneCopoeia (Rockville, MD), and was used according to the manufacturer’s protocol.

Chemical compound screening

The SCADS Inhibitor Kit, an inhibitor set consisting of 380 chemical inhibitors with different targets, was provided by the Screening Committee of Anticancer Drugs, supported by a Grant-in-Aid for Scientific Research on Innovative Areas, Scientific Support Programs for Cancer Research, from The Ministry of Education, Culture, Sports, Science and Technology, Japan. The screening was performed by high-throughput reporter assays. LX2 cells were transfected with a SRF-driven firefly luciferase construct, a RASAL1-expressing plasmid, and pGL4-TK, a Renilla luciferase construct as an experimental control. At 24 h after transfection, cells were seeded in 96-well plates and 5 µM of inhibitors in DMSO were added for 24 h. Luciferase values were measured using a GLOMAX 96-well microplate luminometer (Promega) and relative values were calculated by adjusting the average values of DMSO-treated control cells to 1.0.

Immunocytochemistry

Cells were seeded in the chamber slides (Nalge Nunc, Naperville, IL). The next day, cells were fixed with 4% paraformaldehyde and permeabilized with 0.5% Triton-X100. Fixed cells were incubated with primary antibodies for 60 min at room temperature, followed by incubation with Alexa Flour 488- or 555-conjugated secondary antibodies (Invitrogen, Carlsbad, CA) for 60 min at room temperature. Slides were mounted using VectaShield with DAPI (Vector Labs, Burlingame, CA). To examine the effects of

TGF-β, cells were incubated with serum-free DMEM for 24 h and treated with 10 ng/mL TGF-β for 60 min before fixation.

Immnohistochemistry

Immnohistochemistry was performed as described previously (3). The area of fibrosis was determined using the ImageJ software.

Ras, RhoA, and Gαi activities

Ras and RhoA activities were measured using the Ras and RhoA Activation Pull-down Biochem Kit from Cytoskeleton (Denver, CO) according to the manufacturer's protocols. Briefly, after washing the cells with ice cold PBS, they were dissolved with cell lysis buffer and harvested using a cell scraper. Cell lysates were transferred to sample tubes and centrifuged (10,000 rpm, 4°C, 2 min). GST-tagged Ras binding domain (RBD) of Raf protein beads for Ras activity and GST-tagged Rhotekin protein beads for RhoA activity were added to the equivalent protein amounts of lysate and incubated at 4°C on a rotator for 1 h. The beads were pelleted by centrifugation (3,000*g*, 1 min, 4°C), and were washed with 500 μL of wash buffer and pelleted by centrifugation (3,000*g*, 3 min, 4°C). After the supernatant was removed carefully and 2× sample buffer was added, the beads samples were boiled for 2 min. Activities of Gαi were measured using the Gαi activation kit from Abcam based on immunoprecipitation using an anti-active Gαi monoclonal antibody according to the manufacturer's protocol. The amounts of GTP-bound Ras and RhoA, and active Gαi proteins, were determined by Western blotting using the specific antibodies provided.

PKA and PKC kinase assay

PKA and PKC activities were determined using PKA and PKC kinase activity assay kits obtained from Abcam (#ab139435 and #ab139437, respectively) based on ELISA, according to the manufacturer's protocol. Briefly, wells containing PKA or PKC substrate peptides were soaked with Kinase Assay Dilution Buffer for 10 min at room temperature and the buffer was aspirated. Samples were added to the appropriate wells and diluted ATP was added to each well to initiate the reaction. The plate was incubated at 30°C for 90 min, and then the liquid was aspirated to stop the reaction. Antibodies against phosphor-specific substrate peptides were added to each well and incubated at room temperature for 60 min, and then liquid was aspirated from all wells. Wells were washed with wash buffer four times. Anti-rabbit IgG:HRP conjugate was added to each well and the plate was incubated at room temperature for 30 min. Wells were then washed. TMB Substrate Solution was added to each well and the plate was incubated at room temperature for 30 min. The Stop Solution was added to each well and the absorbance at 450-nm wavelength was measured using a microplate reader.

Quantitative real-time PCR (qRT-PCR)

The Fibrosis RT^2 Profiler PCR Array was obtained from QIAGEN (Hilden, Germany) and used for qRT-PCR analysis. qRT-PCR was performed as described previously (3). Briefly, total RNA was isolated from cells using TRIzol Reagent (Invitrogen, Carlsbad, CA). cDNA was synthesized from RNA using the SuperScript III First-Strand Synthesis System (Invitrogen). Quantitative PCR was performed using the TaqMan gene expression system with Sybr Green (Applied Biosystems, Foster City, CA). All values were normalized to the level of mRNA of the housekeeping gene, GAPDH, and the

relative expression was calculated according to the $\Delta\Delta C_T$ method: $\Delta\Delta C_T = \Delta C_{Tsample} - \Delta C_{Tgapdh}$.

Cell growth assays

Cells were plated in 48-well plates at a density of 1×10^4 per well and cultured in DMEM supplemented with 2% FBS. After incubation for 1-7 days, viable cells were quantified using the Cell Counting Kit-8 (Dojindo, Rockville, MD) with 2-(2-methoxy-4-nitrophenyl)-3-(4-nitrophenyl)-5-(2,4-disulfophenyl)-2H-tetrazolium monosodium salt (WST-8) as a substrate, according to the manufacturer's protocol. Absorbances at 450 nm were measured using a microplate reader (Model 680; Bio-Rad, Hercules, CA).

SUPPLEMENTARY REFERENCES

1. Xu L, Hui AY, Albanis E, Arthur MJ, O'Byrne SM, Blaner WS, Mukherjee P, et al. Human hepatic stellate cell lines, LX-1 and LX-2: new tools for analysis of hepatic fibrosis. Gut 2005;54:142-151.
2. Ohta M, Seto M, Ijichi H, Miyabayashi K, Kudo Y, Mohri D, Asaoka Y, et al. Decreased expression of the RAS-GTPase activating protein RASAL1 is associated with colorectal tumor progression. Gastroenterology 2009;136:206-216.
3. Takata A, Otsuka M, Yoshikawa T, Kishikawa T, Hikiba Y, Obi S, Goto T, et al. MicroRNA-140 acts as a liver tumor suppressor by controlling NF-κB activity by directly targeting DNA methyltransferase 1 (Dnmt1) expression. Hepatology 2013;57:162-170.
4. Kojima K, Takata A, Vadnais C, Otsuka M, Yoshikawa T, Akanuma M, Kondo Y, et al. MicroRNA122 is a key regulator of alpha-fetoprotein expression and influences the aggressiveness of hepatocellular carcinoma. Nat Commun 2011;2:338.
5. Manabe I, Owens GK. CArG elements control smooth muscle subtype-specific expression of smooth muscle myosin in vivo. J Clin Invest 2001;107:823-834.

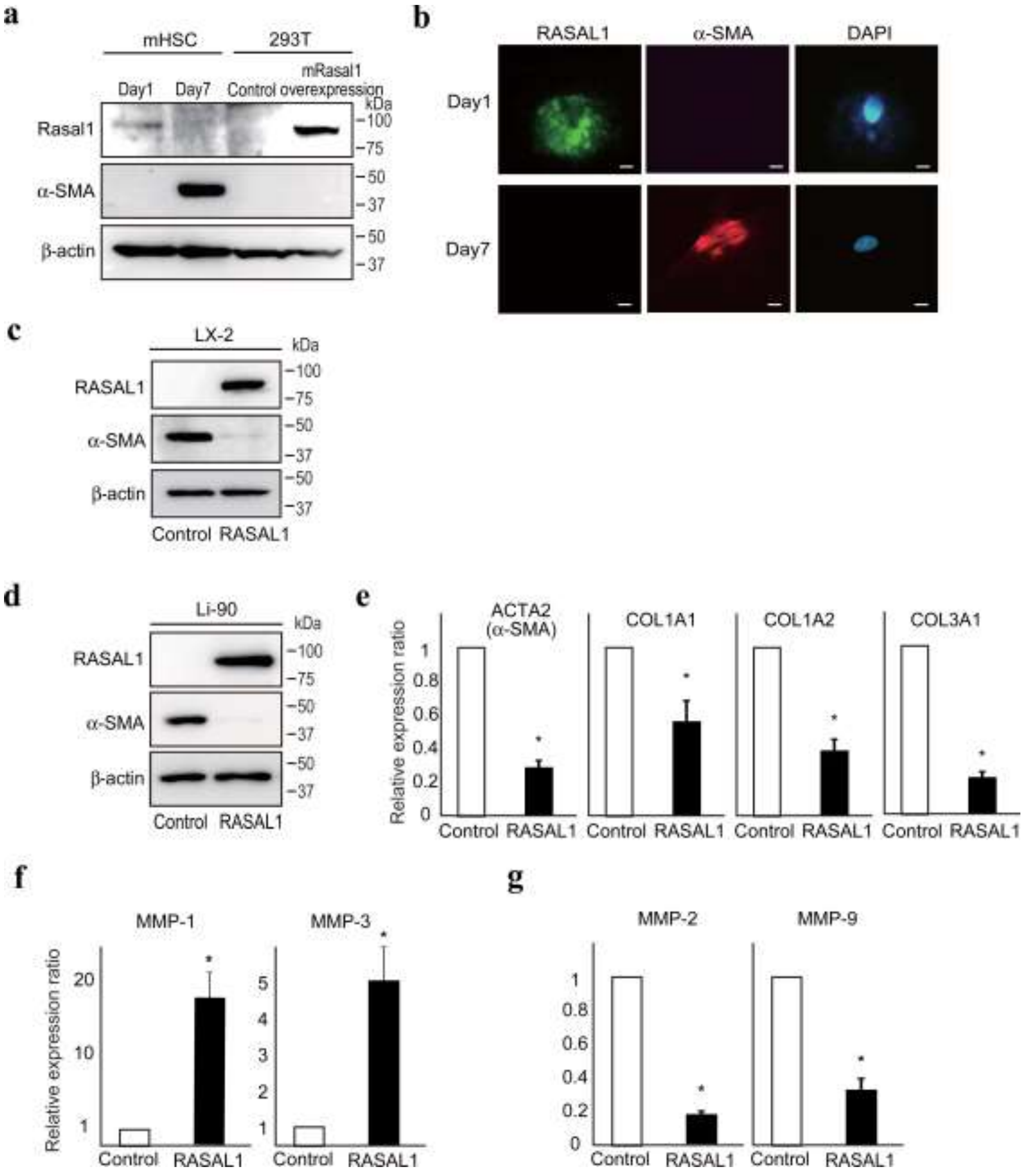

Supplementary Figure S1. **RASAL1 suppresses the activity of hepatic stellate cells**

a, Protein levels of α-SMA and RASAL1 in mouse primary HSCs during *in vitro* culture were assessed by Western blotting. 293T cell lysates transiently transfected with mouse RASAL1-expressing plasmid were used as a positive control. Representative results from two independent experiments are shown. **b**, Protein expression levels of α-SMA (red) and RASAL1 (green) in mouse primary HSCs during *in vitro* culture were

assessed by immunocytochemistry. Representative results from three independent experiments are shown. Scale bar, 10 μm. **c**, Protein levels of α-SMA in LX2 cells with forced RASAL1 expression were determined by Western blotting. Representative results from three independent experiments are shown. **d**, Protein expression levels of α-SMA in control and forced RASAL1-expressing Li-90 human HSCs were assessed by Western blotting. Representative results from three independent experiments are shown. **e**, Expression levels of fibrosis-related gene transcripts were assessed by quantitative RT-PCR. Values shown represent mRNA expression levels in RASAL1-expressing cells relative to those in control Li-90 cells. Data represent the means ± SD of three independent experiments. *, $p < 0.05$ (*t*-test). **f**, **g**, Expression levels of MMP-related gene transcripts were assessed by quantitative RT-PCR. Values shown represent mRNA expression levels in RASAL1-expressing LX2 cells relative to those in control LX2 cells. Data represent the means ± SD of three independent experiments. *, $p < 0.05$ (*t*-test).

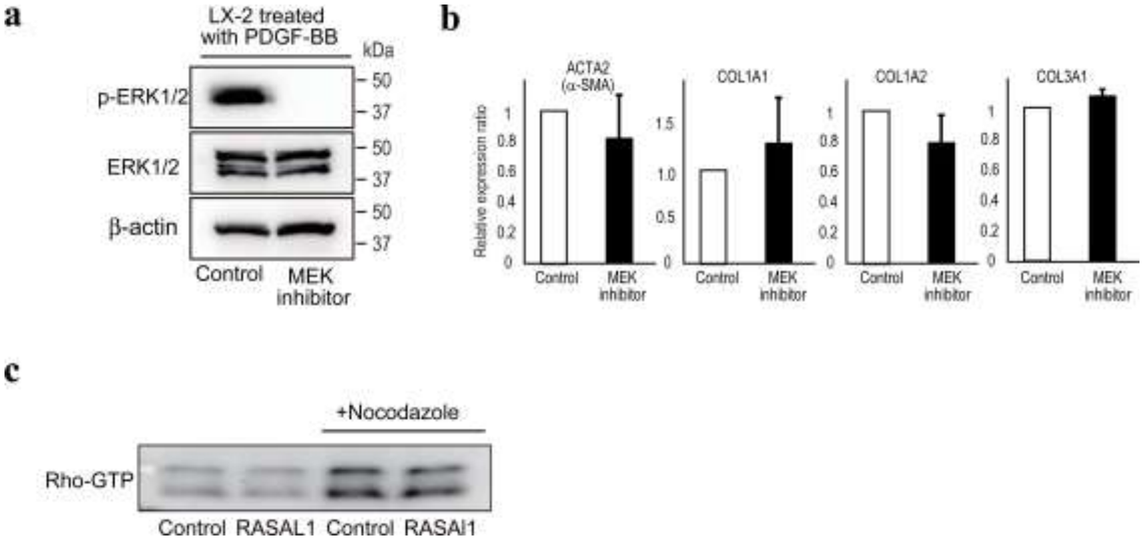

Supplementary Figure S2. **Rho and MAPK activities are independent of the effects of RASAL1 on fibrotic changes in HSCs**

a, A MEK inhibitor significantly inhibited Erk1/2 phosphorylation after treatment with PDGF for 1 h, resulting in activation of the MAPK pathway in LX2 cells. Representative results of three independent experiments are shown. **b**, A MEK inhibitor had no effect on the expression of fibrotic gene transcripts in LX2 cells. The expression levels of fibrosis-related gene transcripts were assessed by quantitative RT-PCR. Values shown represent mRNA expression levels in LX2 cells with or without MEK inhibitor treatment for 24 h. **c,** Rho activity was determined by comparing the amounts of active GTP-bound Rho (Rho-GTP) that bound with Rhotekin between control and RASAL1-expressing LX2 cells. Nocodazole treatment for 1 h was used as a positive control for the activation of Rho function. Representative results of five independent experiments are shown.

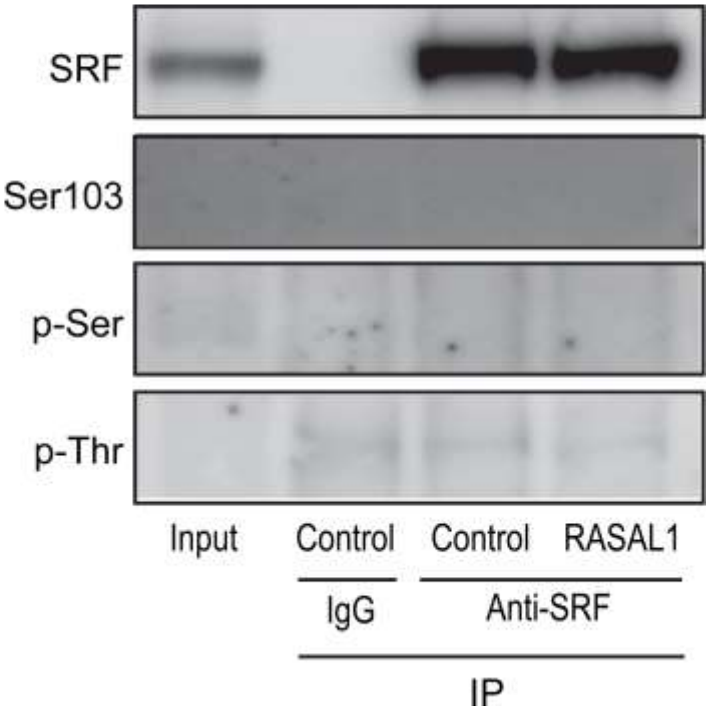

Supplementary Figure S3. **Phosphorylation status of SRF is not changed by RASAL1 expression**

SRF protein in control (Control) and RASAL1-overexpressing (RASAL1) LX2 cells

was immunoprecipitated using anti-SRF antibodies. Normal rabbit IgG was used as a negative control for immunoprecipitation. Five percent of cell lysates was used as a loading control (Input). Immunoprecipitates were blotted using anti-SRF, anti-serine 103 phosphorylation of SRF, anti-serine, and anti-threonine antibodies. Representative results of two independent experiments are shown.

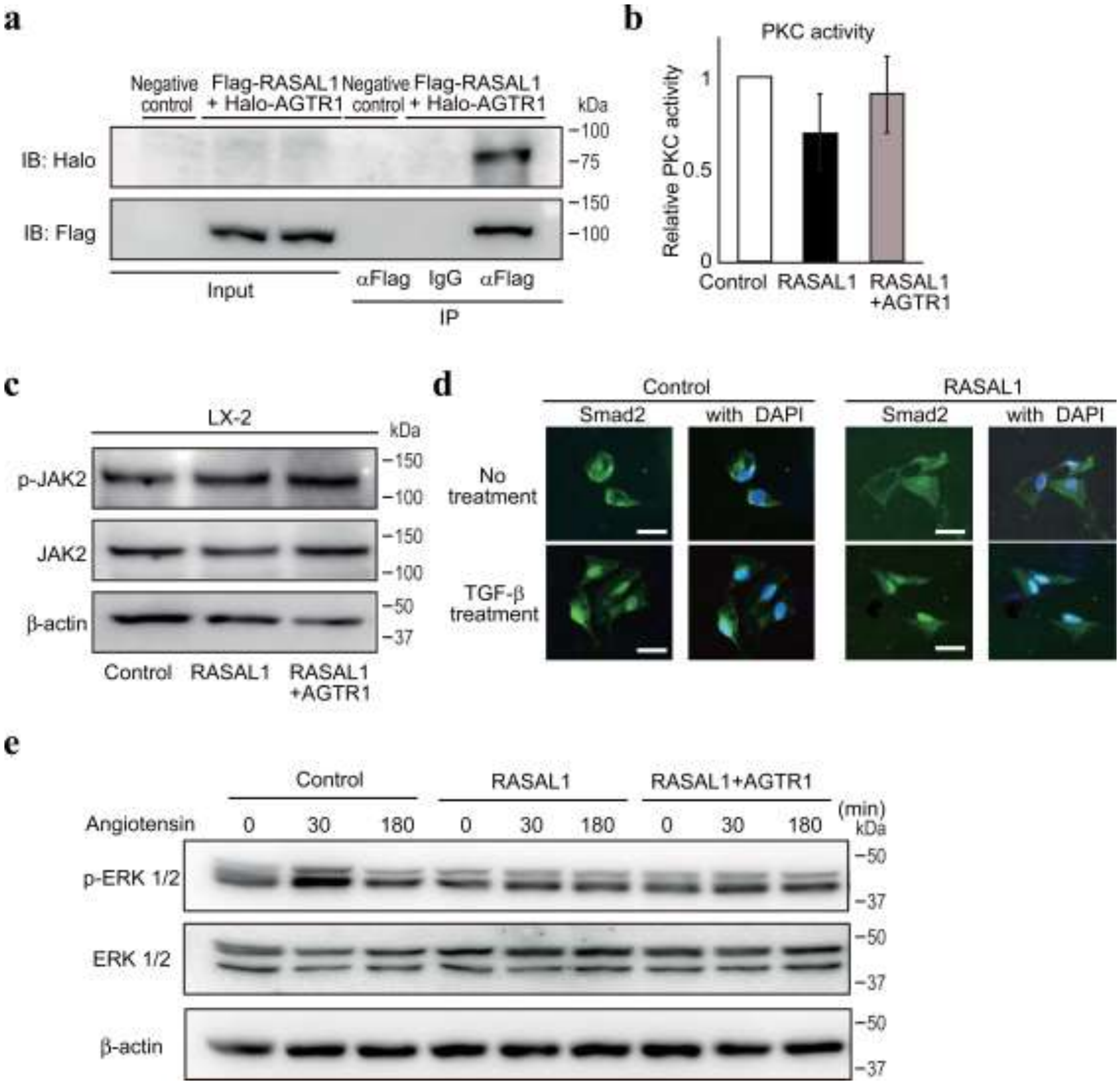

Supplementary Figure S4. **RASAL1 interacts with AGTR1**

a, LX2 cells were transfected with a Flag-tagged RASAL1-expressing plasmid and a Halo-tagged AGTR1-expressing plasmid. Cell lysates without transfection were used as

a control. Flag-tagged RASAL1 was immunoprecipitated using anti-Flag agarose. Agarose-conjugated anti-mouse IgG was also used as a negative control. Co-precipitated proteins were blotted using antibodies against the indicated proteins. Of the total cell lysates, 5% were loaded as an input control. Representative results from three independent experiments are shown. **b**, Activities of PKC in control, RASAL1-expressing, and RASAL1 + AGTR1-coexpressing LX2 cell lysates were measured by ELISA using phosphor-specific antibodies against PKC substrate peptides. Data represent the means ± SD of three independent experiments. **c**, RASAL1 had no effect on the phosphorylation of JAK2. The JAK2 phosphorylation status in control (Control), RASAL1-expressing (RASAL1), and RASAL1 and AGTR1 co-expressing (RASAL1 + AGTR1) LX2 cells was determined by Western blotting. Representative results from three independent experiments are shown. **d**, Smad2 entered the nuclei under TGF-β treatment regardless of RASAL1 expression in LX2 cells, as determined by immunocytochemistry. Smad2 was stained green and nuclei were stained with DAPI, in blue. Representative results from two independent experiments are shown. Scale bar, 10 μm. **e**, The Erk1/2 phosphorylation status following angiotensin II treatment was determined by Western blotting. While control LX2 cells showed increased Erk1/2 phosphorylation following angiotensin II treatment, this was inhibited by RASAL1 overexpression, irrespective of AGTR1 expression. Representative results from three independent experiments are shown.

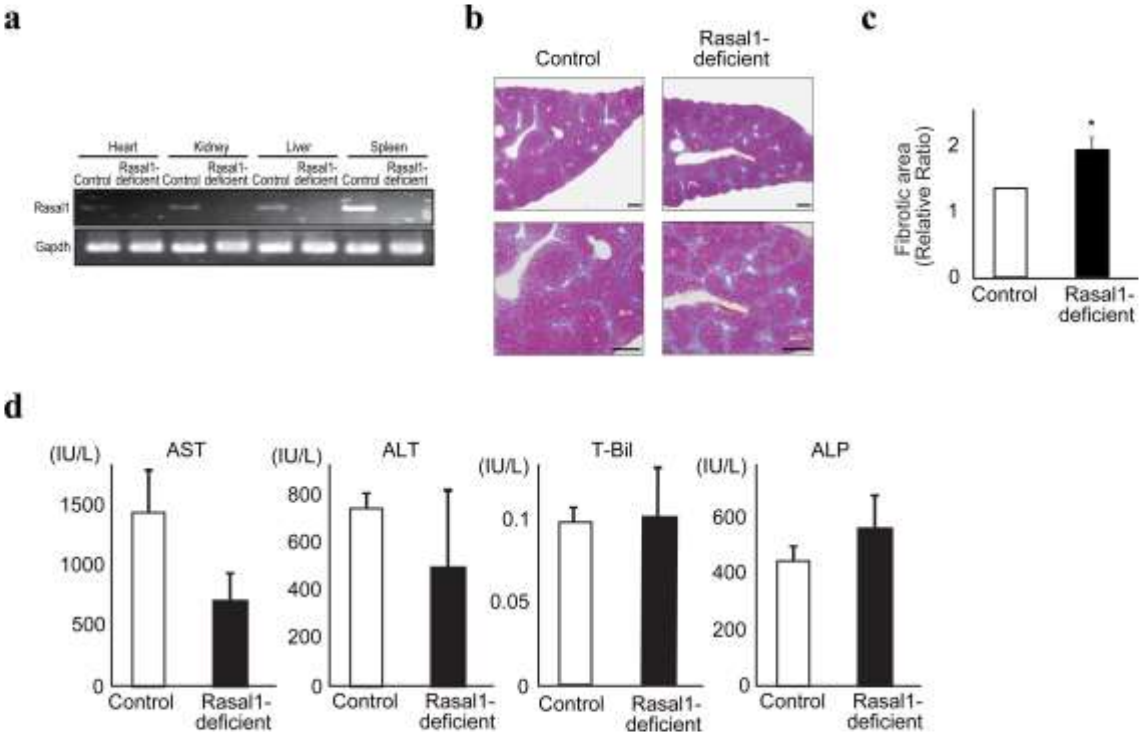

Supplementary Figure S5. **RASAL1-deficient mice are more prone to liver fibrosis**

a, Lack of RASAL1 mRNA expression was confirmed in several tissues of RASAL1-deficient mice. Total RNA from various mouse tissues was extracted and used for determination of Rasal1 transcript levels by RT-PCR. GAPDH transcripts served as an endogenous control. **b**, Liver histology images with Masson's trichrome staining after the induction of experimental liver fibrosis by TAA are shown. While both control and RASAL1-deficient mice showed liver fibrosis (stained in blue), the degree of fibrosis was significantly greater in RASAL1-deficient mice. Representative results from four mice in one group are shown. Scale bar, 500 μm. **c**, Quantitative liver fibrotic areas in control and RASAL1-deficient mice determined using the ImageJ software. Data represent the means ± SD of four mice in one group. *, $p < 0.05$ (*t*-test). **d**, The degree of liver inflammation was similar in control and RASAL1-deficient mice. Biochemical parameters reflecting liver inflammation were measured in sera at 8 weeks

after TAA treatment. Data represent the means ± SD of four mice in one group. *, $p < 0.05$ (*t*-test).

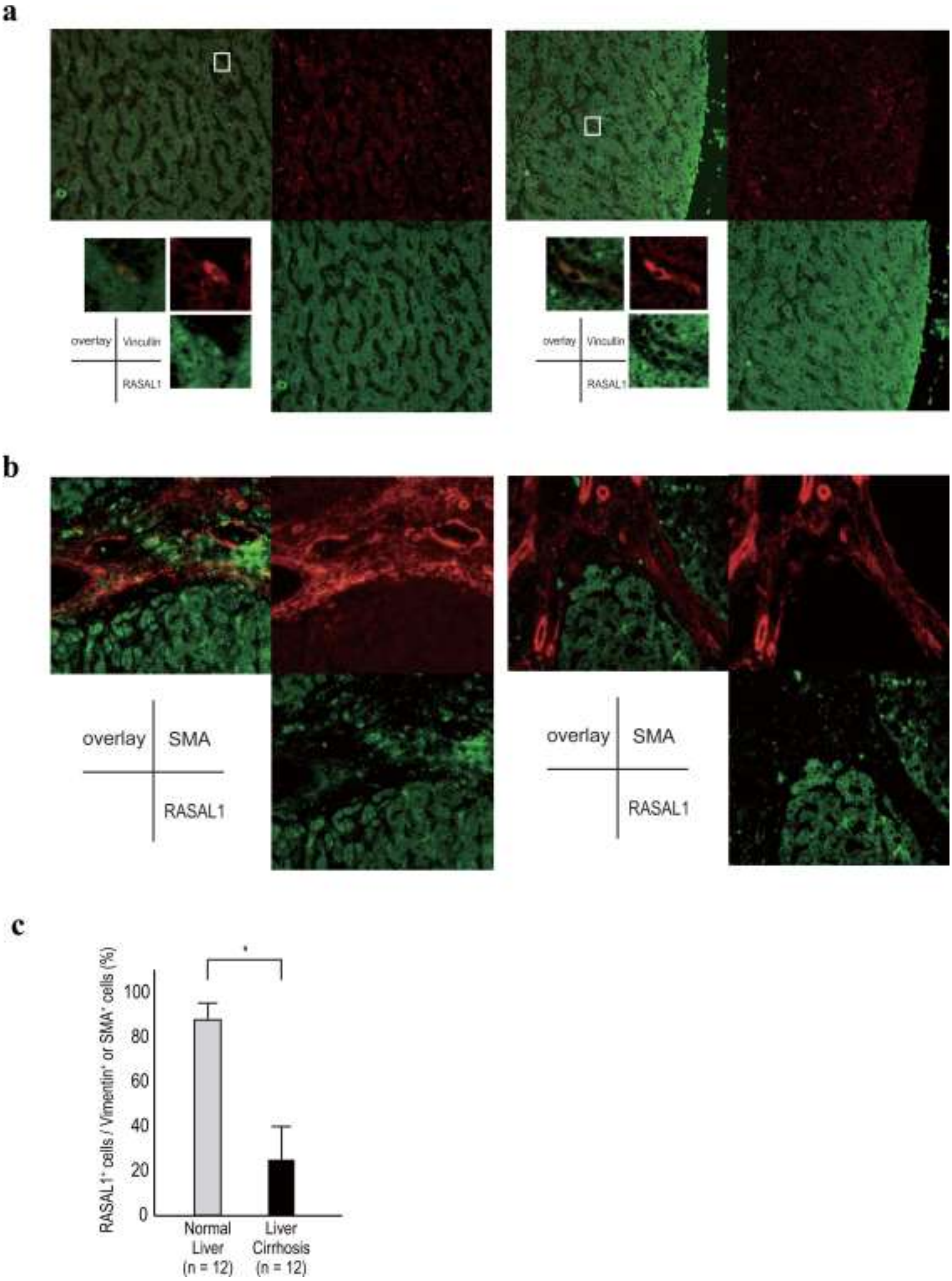

Supplementary Figure S6. **RASAL1 expression is down-regulated in human**

activated HSCs

a, b, Resting HSCs express RASAL1 but activated HSCs do not in human liver tissues. RASAL1 protein was immunostained in green. Resting HSCs were marked by staining Vimentin (a resting marker) in red in normal liver tissues (a). Activated HSCs were by SMA (an activating marker) in red in fibrotic liver tissues (b). Please note: hepatocytes also express RASAL1 but its expression levels are unchanged in normal (a) and fibrotic liver (b). Regions in the white rectangles in upper images are enlarged below. **c,** The percentage of RASAL1 expressing HSCs in resting (Vimentin+) in normal liver and activating (SMA+) state in liver cirrhosis tissues. Data represent the means ± SD of 12 samples in one group. *, $p < 0.05$ (*t*-test).
